# Knowledge and perception of end-of-life bioethics among medical students. A comparative study between the University of Verona (Italy) and the University of Halle (Germany)

**DOI:** 10.1186/s12910-026-01507-2

**Published:** 2026-07-17

**Authors:** Sara Patuzzo Manzati, Michele Piazza, Simone Garzon, Maria Bencivenga, Giovanni de Manzoni, Alexander Kremling, Christian König, Jan Schildmann

**Affiliations:** 1 Department of Surgery, Dentistry, Paediatrics and Gynaecology, University of Verona, Verona, Italy; 2 Medical Faculty of Martin, Institute for History and Ethics of Medicine, Interdisciplinary Centre for Health Sciences, Luther University Halle-Wittenberg, Halle (Saale), Germany; 3 Institut für Geschichte und Ethik der Medizin, Profilzentrum Gesundheitswissenschaften, Medizinische Fakultät der Martin-Luther-Universität Halle-Wittenberg, Magdeburger Straße 8, Halle (Saale), 06112 Germany; 4 https://www.umh.de/igem

## Abstract

**Background:**

End-of-life care raises complex ethical and legal questions shaped by national frameworks, cultural contexts, and personal beliefs. This comparative study explores how fifth-year medical students in Verona (Italy) and in Halle (Germany) understand and evaluate key end-of-life practices, including advance directives, withholding or withdrawing life-sustaining treatment, palliative care, medically assisted suicide, and life termination through drugs. The study also examines whether religiosity influences ethical orientations.

**Methods:**

A structured questionnaire consisting of 16 closed-ended items and one open question was administered to fifth-year medical students at the University of Verona and the University of Halle during the 2024–2025 academic year. Items assessed knowledge of legal frameworks, ethical attitudes, personal experiences with dying patients, and perceived educational preparedness. Descriptive statistics, cross-tabulations, and chi-square tests were used to compare cohorts, with statistical significance set at *p* < 0.05.

**Results:**

A total of 259 valid responses were analyzed. Students in both countries showed strong consensus regarding the legitimacy and clinical usefulness of advance directives and the central role of palliative care. Acceptance of withholding or withdrawing treatment was high in both cohorts, though slightly higher in Germany. Marked differences emerged regarding life termination through drugs, with Italian students expressing more permissive and polarized positions and German students showing more moderate and cautious responses. Attitudes toward medically assisted suicide were broadly permissive in both groups, with religiosity emerging as a consistent predictor of more restrictive positions, particularly in Italy.

**Conclusions:**

While future physicians in both countries share core commitments to patient autonomy and palliative care, significant differences persist in their evaluation of practices intended to hasten death. These findings suggest that ethical orientations at the end of life are shaped not only by shared professional principles but also by national cultural environments and personal belief systems. Comparative research among medical students offers valuable insight into how medico-legal contexts and education influence emerging professional ethics.

**Supplementary Information:**

The online version contains supplementary material available at 10.1186/s12910-026-01507-2.

## Introduction

This study compares medical students from the Universities of Verona (Italy) and Halle (Germany) to explore knowledge and ethical attitudes toward key end-of-life practices, including Advance Directives (ADs), Withdrawing/withholding Life-Sustaining Treatment (WLST), Palliative Care (PC), Medically Assisted Suicide (MAS), and active Life Termination Through Drugs (LTTD). These two centres were selected to reflect distinct medico-legal and cultural contexts within Europe.

In Germany, historical memory of Nazi medicine has fostered enduring ethical and legal caution toward practices involving the intentional ending of life, influencing both regulation [1, 2] and terminology, with the term “Euthanasie” largely avoided in current legal and public discourse [3, 4]. Beyond this historical dimension, the German socio-cultural context is generally characterized by a predominantly secular and pluralistic society; however, institutional religion still retains a role in public ethical debates, and Christian traditions continue to inform certain aspects of the moral discourse on end-of-life issues.

In the Italian context, the bioethics of the sanctity of life is deeply rooted and historically reinforced by the strong influence of Catholic moral theology, contributing to a socio-cultural framework in which the protection of life has traditionally represented a central ethical value. At the same time, Italy has experienced a gradual pluralization of moral perspectives, reflected in recent legislative developments and case law that have increasingly recognized individual autonomy even at the end of life.

ADs are legal instruments through which competent individuals express healthcare preferences in anticipation of future decisional incapacity [5–8]. WLST refers to discontinuing (withdrawing) or not beginning (withholding) interventions necessary to sustain life [9–11]. PC aims at treating pain and relieving suffering for patients with serious illnesses [12–15]. LTTD and MAS are often jointly discussed in the bioethical literature under the umbrella term Medically Assisted Death (MAD) [16, 17]. LTTD involves the direct administration of a lethal substance by a physician with the intention of causing death. MAS involves a physician providing access to lethal medication, to end one’s life, while the patient retains control over and performs the final life-ending act [18, 19].

Given the well-documented role of religion in shaping moral attitudes toward life, suffering, and death, religious affiliation was included as a variable to better interpret potential differences between or within the two cohorts.

The students participating in the study are enrolled in the fifth year of their degree program. This stage of their training was selected as it is considered crucial: they have completed most of their medical-scientific and medical ethics education and are beginning to be involved in clinical settings where they encounter patients with serious illnesses, terminal diagnoses, and end-of-life situations [20]. Therefore, these students represent a cohort at the threshold between theoretical knowledge and clinical practice, and assessing their perceptions may be particularly insightful, as their attitudes have not yet become fully crystallized [21].

## Legal frameworks in Italy and Germany

The legal frameworks governing end-of-life practices in Italy and Germany currently exhibit significant areas of convergence in terms of the rights recognized and the instruments made available to individuals within care pathways. These convergences include the recognition of the right to refuse or discontinue medical treatments, including WLST; the availability and binding nature of ADs and advance care planning tools; the patient’s right to receive clinically appropriate PC; the gradual legitimization of MAS through constitutional jurisdiction interventions; and the prohibition of LTTD.

### Italy

In Italy, freedom of treatment, and therefore the individual’s right to accept or refuse medical interventions, has been recognized since the Constitution (1948) and subsequently reaffirmed by Law 833/1978, as well as by the Code of Medical Ethics from its first republican edition (1958) through to the currently valid version (2014) [22]. Law 219/2017 reaffirms the individual’s right to accept or refuse the initiation or continuation of a physician’s proposed treatment, explicitly including the right to refuse or discontinue LST (Art. 1, paragraph 5) [8, 23]. In this way, consent and dissent are configured as legally binding expressions of the patient’s will, to which the physician is obliged to adhere. Such will may be expressed either contemporaneously (Art. 1) or in advance, through ADs (Art. 4) and Advance Care Planning (ACP, Art. 5).

Law 219/2017 establishes the prohibition of futile care (Art. 2, para. 2) and reaffirms the patient’s right to receive clinically appropriate PC (Art. 2, para. 1), as already provided for by Law 38/2010, including continuous deep palliative sedation in association with symptom management when suffering is refractory to conventional medical treatments (Art. 2, para. 2).

Regarding MAD, there are no laws in Italy regulating euthanasia or MAS. Consequently, these two practices are respectively equated with the crimes of homicide or consensual homicide if the act is carried out at the person’s request (Penal Code, Art. 579), and assistance to suicide (Penal Code, Art. 580). While euthanasia remains prohibited despite a referendum attempt indicating popular support for its legalization (2001–2002), MAS has been legitimized through Constitutional Court interventions in the Antoniani/Cappato case [24]. In ruling No. 242/2019, the Court outlined the conditions for its lawful practice: the individual must possess the capacity to make free and informed decisions, declare intolerable suffering, and present an irreversible condition along with LST [25]. In ruling No. 135/2024, the Court clarified that LST should be understood as those procedures whose omission or discontinuation would result in the patient’s death within a short period, including interventions that could also be performed by family members or caregivers, such as manual bowel evacuation, urinary catheterization, or mucus aspiration from the airways. Although MAS has been *de facto* permitted within the Italian National Health Service since 2019, Parliament has not yet enacted specific legislation on the matter, leaving Hospitals and regional Authorities to self-regulate regarding access procedures, verification of eligibility, and implementation [26].

### Germany

As in the Italian context, the German legal framework for end-of-life practices is based on the recognition of informed consent and individual will as decisive criteria for the lawfulness of medical interventions, both when expressed contemporaneously and in advance. Informed consent is constitutionally protected as an expression of the “General right of personality” and the principle of individual self-determination, derived from Article 2, paragraph 1 (“Free development of personality”), in conjunction with Article 1, paragraph 1 (“Human dignity”) of the Basic Law (*“*Basic Law”).

ADs constitute legally binding instruments regulated by the German Civil Code (1827 BGB – “Individual provision”) [27].

Under the *“*Hospice and palliative care act” (2015) and in particular § 132 g, “Health care planning for the last phase of life”, ACP and associated counselling is funded for example for persons in nursing homes.

As in the Italian case, regarding WLST, German law recognizes the lawfulness of withholding/withdrawing LST in accordance with the patient’s will, whether expressed contemporaneously or in advance. The jurisprudence of the German Federal Court of Justice has clarified that the patient’s will constitutes the legally decisive criterion for justifying the cessation of LST, regardless of the distinction between withholding and active withdrawal of treatment [28, 29].

On the issue of MAD, understood here as an analytical category rather than a positive legal notion, the administration of drugs with the intent to directly cause the patient’s death at their request remains illegal under Sect. 216 of the German Criminal Code [30].

Unlike the Italian context, assisted suicide (AS) underwent a constitutional legal shift with the ruling of 26 February 2020 by the Federal Constitutional Court, which declared Sect. 217 StGB, which had been introduced only five years earlier, unconstitutional.

 [31]. In this decision, the Court recognized a constitutional right to self-determined death, grounded in the general right of personality (Art. 2, paragraph 1, in conjunction with Art. 1, paragraph 1 of the Basic Law), encompassing both the freedom to end one’s own life and to seek and use assistance voluntarily offered by third parties, without the state having the authority to scrutinize the subjective reasons underlying such a choice [32, 33]. The federal constitutional court defined “Free responsibility”, which encompasses decisional capacity, informed decision-making, voluntariness and stability of the wish to die, as the only eligibility criterion for lawful AS [34].

Following the 2020 decision, the German legislature attempted to introduce statutory regulation of access to MAS; however, in July 2023 the Federal Parliament rejected two competing legislative proposals, leaving the country without a comprehensive legislative framework [35]. This has resulted in a situation in which (M)AS is, in principle, legally permissible. However, there is a lack of an operationalization of the criteria established by the federal constitutional court. No unified procedural framework exists, and access may depend on practical factors, including physician availability, the policies of suicide assistance organizations, and restrictions related to the prescription and access to controlled medications [36].

## Objectives

The present study aims to explore the knowledge, perception, and ethical orientation of fifth-year medical students regarding key bioethical issues in end-of-life care. A structured questionnaire was administered to assess their understanding of current legislation and their personal opinions and attitudes toward core topics such as accompaniment at the end of life, ADs, WLST, PC, and MAD, including LTTD and MAS.

The research was conducted at two academic institutions, the University of Verona (Italy) and the University of Halle (Germany), to perform a comparative analysis between distinct educational, cultural, and legal contexts.

A further objective was to examine the role of religious affiliation as a potential factor influencing students’ responses, thereby assessing how personal belief systems interact with ethical reasoning and professional attitudes toward end-of-life decision-making.

## Methods

### Questionnaire composition

The questionnaire was developed by a multidisciplinary team of bioethicists, clinicians, and medical educators from both institutions. Development followed a systematic process including: (1) literature review of existing validated instruments on medical students’ end-of-life ethical attitudes; (2) expert content validation through iterative team review; (3) alignment with current national legal frameworks for end-of-life practices in Italy and Germany. The instrument was informally pre-tested by the research team for clarity prior to administration.

The questionnaire comprises 16 single-choice items and one open-ended question, designed to investigate medical students’ knowledge, opinions, and personal experiences regarding key bioethical issues related to end-of-life care. The first section explored students’ direct or indirect experiences with dying patients, within family contexts, clinical rotations, or other settings, as well as any exposure to patient requests for MAD.

The second section focused on ADs, assessing students’ awareness of their legal status in their respective countries and their views on the ethical and clinical relevance of ADs as instruments for expressing patient preferences and guiding medical decisions in terminal situations.

The third section addressed WLST and PC, investigating students’ opinions on the right to refuse life-prolonging interventions, the ethical legitimacy of treatment withdrawal under specific conditions, and the perceived importance of integrating palliative care within clinical practice.

The fourth and fifth sections explored MAD, including LTTD and MAS, by probing students’ knowledge of the relevant legal frameworks and their personal ethical positions, measured through an agreement–disagreement Likert scale. The term “Active life termination” was used as an umbrella expression referring to euthanasia and medically assisted suicide. No formal operational definition was provided to participants beyond the wording of the questionnaire items.

The sixth section focused on medical education, asking students to self-assess their preparedness, at the conclusion of their training, to address ethical issues related to end-of-life care.

Finally, the questionnaire collected sociodemographic data (gender, age, and religious affiliation) to enable the analysis of possible correlations between personal characteristics and ethical orientations across the different thematic areas.

### Approval and administration

At the University of Verona, the questionnaire and its administration procedure were approved by the President of the School of Medicine and Surgery. At the University of Halle, approval was granted by the research ethics commission of the Medical Faculty (RegNr. 2024 − 184).

In both institutions, participants received a brief explanation of the study’s objectives and procedures before completing the questionnaire. Fifth-year students enrolled in the Degree Course in Palliative Medicine (Halle/Saale) and Medicine and Surgery (Verona) during the 2024–2025 academic year were invited to take part voluntarily, following the provision of informed consent. The questionnaire was administered in paper format.

### Scales and measurement of variables

Personal experience with end-of-life situations was assessed through a dichotomous response option (“yes” or “no”). For affirmative answers, participants were further asked to rate the emotional intensity of the experience on a 3-point scale (0 = “not very intense,” 1 = “intense,” 2 = “very intense”).

Knowledge of current legislation was measured using either a 2-point (“yes”/“no”) or a 3-point (“yes,” “no,” “I don’t know”) response format, depending on the item, to capture both certainty and awareness gaps regarding legal norms.

Attitudes toward the limitations of medical treatment and the ethical permissibility of ending life at the patient’s request were assessed through a 5-point Likert scale (1 = “not at all true of me” to 5 = “extremely true of me”), enabling a graded evaluation of moral orientation and agreement.

Finally, participants evaluated their perceived educational preparedness to address ethical issues in end-of-life care using a 6-point Likert scale (0 = “not at all prepared” to 5 = “excellently prepared”).

### Data processing and management

Descriptive statistical analyses and cross-tabulations were performed using the statistical software SPSS for Windows, version 26.0 (IBM Corp., Chicago, IL, USA). Chi-square tests were used to identify bivariate associations between categorical variables (e.g., site, religiosity, and responses). In addition, bivariate logistic regression models were applied to assess whether site or religious affiliation independently predicted specific outcomes. This analytical approach was chosen to allow a transparent and straightforward comparison between cohorts and to explore associations between categorical variables without overfitting the data. The level of statistical significance was set at *p* < 0.05.

## Results

The results presented below are to be considered exploratory in nature, taking into account the characteristics of the study design and the sample size, and are intended to inform and generate hypotheses for future research.

Of the 278 completed questionnaires returned, 19 or 6.8% (12/95 [12.6%] Halle, 7/183 [3.8%] Verona) were excluded due to incompleteness (> 20% missing) [37, 38], yielding 259 valid responses (93.2% completion rate of returned questionnaires).

### Sociodemographic characteristics (Table 1)

**Table 1:** Sociodemographic characteristics. Participant characteristics by site (Verona and Halle) are presented as numbers (with group percentages) for categorical variables and as means (with standard deviations, SD) for numerical variables

	Verona 176	Halle 83	Total 259
Age, years (SD)	24.31 (1.86)	25.64 (3.29)	24.71 (2.46)
minimum	22	21	21
maximum	35	36	36
Gender, *n* (%)			
male	68 (38.6)	25 (30.1)	93 (35.9)*
female	108 (61.4)	57 (68.7)	165 (63.7)*
not specified	0	1(1.2)	1 (0.4)*

*percentage of the total participants enrolled

The mean age is slightly higher in Halle (25.6 years) than in Verona (24.3 years). Female students represent the majority in both cohorts (61.4% in Verona; 68.7% in Halle). Overall, demographic differences between the two groups are limited. These similarities suggest that observed differences in responses are unlikely to be primarily driven by basic demographic variables, although other unmeasured educational and cultural factors may still contribute.

### Religious affiliation and belief (Table 2)

**Table 2:** Religious affiliation and belief. Religious affiliation and belief by site (Verona and Halle) are presented as numbers (with group percentages)

	Verona 176	Halle 83	Total 259*
Religious *n*, (%)			
yes	89 (50.6)	38 (45.8)	127 (49)
no	85 (48.3)	42 (50.6)	127 (49)
not specified	2 (1.1)	3 (3.6)	5 (1.9)
Religion, *n* (%)			
Christian-Catholic	83 (47.2)	25 (30.1)	108 (41.7)
Christian-Protestant	2 (1.1)	9 (10.8)	11 (4.2)
Muslim	2 (1.1)	0 (0)	2 (0.8)
Other	2 (1.1)	4 (4.8)	6 (2.3)
Atheist/Agnostic	85 (48.3)	42 (50.6)	127 (49.0)
No answer	2 (1.1)	3 (3.6)	5 (1.9)

Religious affiliation is evenly distributed between the two cohorts, with approximately half of respondents identifying as religious (50.6% in Verona; 45.8% in Halle).

In Verona, 83 out of 176 students (47.2%) identify as Catholic, 2 (1.1%) as Protestant, 2 (1.1%) as Muslim, and 2 (1.1%) as belonging to other religions. Eighty-five students (48.3%) describe themselves as atheist or agnostic.

In Halle, 25 out of 83 students (30.1%) identify as Catholic, 9 (10.8%) as Protestant, and 4 (4.8%) as belonging to other religions, while 42 (50.6%) report being atheist or agnostic. The overall distribution thus shows a predominance of Catholic affiliation in Verona and, in Halle, a comparatively higher proportion of Protestant and non-religious students. A small number of respondents in both cohorts do not specify their religious affiliation (1.1% in Verona; 3.6% in Halle).

No statistically significant differences are observed between the two sites.

### Personal experience with end-of-life situations (Q 1.1–1.2) (Table 3)

**Table 3:** Personal experience with end-of-life situations. Personal experience with end-of-life situations by site (Verona and Halle) are presented as numbers of students (with group percentages)

	Verona	Halle	*P* value
Experience	45/176	48/83	< 0.001
Family, *n* (%)	42 (23.9)	43 (51.8)	0.029
not very intense	0	6	
intense	14	9	
very intense	28	28	
Training, *n* (%)	29 (16.5)	46 (55.4)	0.054
not very intense	9	6	
intense	18	29	
very intense	2	11	

Students in Halle report significantly greater exposure to end-of-life situations than those in Verona (57.8% vs. 25.6%; *p* < 0.001). This difference applies to both family experiences (51.8% in Halle vs. 23.9% in Verona; *p* = 0.029) and clinical encounters during training (55.4% vs. 16.5%; *p* = 0.054).

Among those with family experience, both groups report similarly high emotional intensity: 65.1% (28 out of 43) of Halle students and 66.7% (28 out of 42) of Verona students describe the situation as *very intense*. However, less intense experiences are reported only in Halle (14.0% “not very intense” vs. none in Verona). However, these differences should be interpreted with caution, as perceived emotional intensity is a subjective measure and may be influenced by interindividual variability in the appraisal of similar experiences.

Regarding training-related encounters, Halle students more frequently rate their experience as *intense* or *very intense* (86.9%, 40 out of 46) compared with Verona students (69.0%, 20 out of 29).

### Advance directives: validity for expressing a person’s will (Q 2.2) (Table 4)

**Table 4:** Advance Directives (Q 2.2): validity for expressing a person’s will. Validity of Advance Directives as a way of expressing a person’s will at the end of life, by site and religious affiliation. Responses are shown as numbers of students

		religion		*p*-value
		no	yes	
Halle	**no**	2	0	0.173
	**yes**	40	38	
Verona	**no**	3	4	0.711
	**yes**	82	82	

Most students in both cohorts agree that Advance Directives (ADs) represent a valid and ethically legitimate means of expressing a person’s will at the end of life. In Verona, 95.9% answer Yes and 4.1% No; in Halle, 97.5% answer Yes and 2.5% No.

The distribution of responses is nearly identical across sites, with no statistically significant differences between religious and non-religious participants (Halle *p* = 0.173; Verona *p* = 0.711). In Halle, all religious students (38 out of 38, 100%) support the validity of ADs, as do almost all non-religious students (40 out of 42, 95.2%). In Verona, the pattern is equally uniform, with 95.3% of religious and 96.5% of non-religious students expressing agreement.

A bivariate logistic regression was performed to explore whether site or religious affiliation were associated with differences in students’ responses. The analysis did not show statistically significant effects for either variable (site *p* = 0.526; religiosity *p* = 0.750). These results should be interpreted in light of the limited scope of the variables included, as self-reported religious affiliation does not necessarily capture the strength or depth of individual beliefs.

### Advance directives: usefulness for physicians in end-of-life decisions (Q 2.3) (Table 5)

**Table 5:** Advance Directives (Q 2.3): usefulness for physicians in end-of-life decisions. Perceived usefulness for physicians in end-of-life decision-making, by site and religious affiliation. Data are presented as the number of students

		religion		total	*p*-value
		no	yes		
Halle	**no**	1	2	2	0.943
	**yes**	41	74	74	
Verona	**no**	0	21	21	0.157
	**yes**	85	152	152	

A comparable pattern emerges regarding the perceived usefulness of Advance Directives (ADs) for physicians in end-of-life decision-making. In Verona, 98.8% of students consider ADs useful and 1.2% not useful; in Halle, 97.5% consider them useful and 2.5% not useful.

No significant within-site differences are observed by religious identification (Halle *p* = 0.943; Verona *p* = 0.157), and regression analyses confirm that neither site nor religiosity significantly predict students’ evaluations.

### Withdrawing life-sustaining treatment in some clinical situations (Q 3.1) (Table 6)

**Table 6:** Withdrawing Life-Sustaining Treatment in some clinical situations (Q 3.1). Withdrawing life-sustaining treatment in some clinical situations, by site and religious affiliation. Data are presented as the number of students

		religion		total	*p*-value
		no	yes		
Halle	**no**	2	0	2	0.174
	**yes**	38	36	74	
Verona	**no**	5	16	21	0.015
	**yes**	79	73	152	

Students were asked whether they believed that medical intervention should be suspended in some clinical situations, even if it is a life-saving treatment (yes/no response). Views on the ethical acceptability of WLST differ between sites. In Verona, 87.9% of students consider WLST ethically acceptable, either always or in specific circumstances, while 12.1% regard it as never acceptable. In Halle, acceptance is even higher (97.4%), and only 2.6% of respondents reject it entirely.

Religious affiliation significantly affects responses in Verona (*p* = 0.015), with religious students expressing more hesitation: 82.0% of them, compared with 94.0% of their non-religious peers, consider withdrawal ethically permissible. In Halle, no comparable within-site differences are observed (*p* = 0.174), indicating a more uniform ethical stance across groups.

When comparing the two cohorts, site membership significantly influences responses (*p* = 0.033), while religiosity narrowly misses statistical significance as an independent predictor (*p* = 0.066).

### Palliative care as an integral part of medical practice (Q 3.3) (Table 7)

**Table 7:** Palliative Care as an integral part of medical practice (Q 3.3). Withdrawing life-sustaining treatment in some clinical situations, by site and religious affiliation. Data are presented as the number of students

		religion		total	*p*-value
		no	yes		
Halle	**no**	3	5	8	0.349
	**yes**	39	32	71	
Verona	**no**	7	1	8	0.025
	**yes**	78	88	166	

Across both sites, a large majority of students (92.7%) agree that Palliative Care (PC) represents an essential component of medical practice. In Verona, 95.4% of respondents endorse this view, compared with 89.9% in Halle.

Religious affiliation is modestly associated with response patterns in Verona (*p* = 0.025): 98.9% of religious students agree or strongly agree, compared with 91.8% of non-religious students. In Halle, no comparable difference is observed (*p* = 0.349), indicating a broadly shared understanding of the professional and ethical importance of palliative care across groups.

A logistic regression analysis, which assesses whether site or religiosity independently predict the likelihood of a given response, confirms that neither variable exerts a significant independent effect.

### Life termination through drugs: ethical judgment (Q 4.2) (Table 8)

**Table 8:** Life termination through drugs (Q 4.2): ethical judgment. Ethical judgment regarding life termination through drugs, by site and religious affiliation. Data are presented as the number of students

		religion		
		no	yes	*p*-value
Halle	**totally disagree**	0	2	0.016
	**not agree**	4	7	
	**undecided**	1	6	
	**agree**	29	18	
	**totally agree**	8	5	
		*p* = 0.05		
Verona	**totally disagree**	3	3	0.001
	**not agree**	1	2	
	**undecided**	4	10	
	**agree**	26	45	
	**totally agree**	51	29	
		*p* = 0.007		

Significant differences emerge between Verona and Halle regarding students’ ethical evaluation of life termination through drugs (*p* = 0.002). The question asks respondents to indicate their level of agreement with the statement “*Active life termination is never ethically justified*”.

In Verona, 40.5% of students totally disagree and 39.9% disagree with this statement, meaning that 80.4% regard active life termination as ethically acceptable under certain conditions. Nineteen students (11.0%) are undecided, while 8.6% agree or totally agree, expressing opposition to the practice.

In Halle, 15.0% of students totally disagree, 56.3% disagree, 18.8% are undecided, and 10.0% agree. Compared with Verona, students in Halle show a slightly higher proportion of undecided responses, suggesting greater moral caution and less polarization in their ethical stance.

Religious affiliation is significantly associated with students’ responses in both cohorts (overall *p* = 0.010). In Halle, religiosity predicts *how* students distribute their answers across the available options, for instance, whether they choose “disagree” rather than “totally disagree”, a relationship captured by the item-level test (*p* = 0.024). However, when responses are grouped simply as “agreement” versus “disagreement,” this difference is no longer statistically significant (overall *p* = 0.078). In other words, religious and non-religious students in Halle tend to take similar general positions but differ in the *degree* of their ethical endorsement or opposition.

In Verona, by contrast, religiosity predicts both levels of analysis: not only the fine-grained distribution of responses (item-level *p* = 0.002), but also the broader division between agreement and disagreement (overall *p* = 0.042). This means that religious affiliation in Verona is associated with both *whether* students accept active life termination as ethically permissible and *how strongly* they express that position.

Specifically, religious students are less likely to disagree with the statement, indicating a more restrictive ethical position, both in Verona (72.0% of religious vs. 89.3% of non-religious) and in Halle (60.5% vs. 81.0%).

In Verona, this effect is reflected in the distribution of responses. Among religious students, agreement and undecided answers together account for 28.0% of responses, compared with 10.7% among non-religious students. Non-religious students show a higher proportion of responses in the “disagree” and “totally disagree” categories.

In Halle, a similar but less pronounced pattern is observed. Religious students show a higher proportion of responses in the intermediate categories (39.5% vs. 19.0% among non-religious students), while firm agreement or disagreement remains less frequent in both groups. Overall, responses are more evenly distributed across categories.

### Life termination through drugs: legalization in specific situations (Q 4.3) (Table 9)

**Table 9:** Life termination through drugs (Q 4.3): legalization in specific situations. Legalization of life termination through drugs in specific situations, by site and religious affiliation. Data are presented as the number of students

		religion		
		no	yes	*p*-value
Halle	**totally disagree**	0	2	0.016
	**not agree**	4	7	
	**undecided**	1	6	
	**agree**	29	18	
	**totally agree**	8	5	
		*p* = 0.05		
Verona	**totally disagree**	3	3	0.001
	**not agree**	1	2	
	**undecided**	4	10	
	**agree**	26	45	
	**totally agree**	51	29	
		*p* = 0.007		

Support for the legalization of active life termination in specific circumstances is high across both cohorts but shows distinct national and religious patterns. In Verona, 46.0% of students (*n* = 80) totally agree and 40.8% (*n* = 71) agree that active life termination should be legalized in certain cases. In Halle, 16.3% (*n* = 13) totally agree and 58.8% (*n* = 47) agree (*p* < 0.001). This indicates stronger and more polarized endorsement among Italian students, and a more moderate, though still largely favourable, position among German students.

Religious affiliation significantly is associated with responses in both groups (overall *p* = 0.005). In Halle, religiosity correlates with the detailed distribution of answers across response options (item-level *p* = 0.016), that is, whether students tend to choose *“agree”* rather than *“totally agree”*, and shows a marginal effect on overall agreement (*p* = 0.05). This means that religious and non-religious students in Halle generally share a similar orientation toward legalization but differ in the intensity of their endorsement.

In Verona, religion significantly is associated with both the general level of agreement (overall *p* = 0.007) and the specific distribution of responses (item-level *p* = 0.001). In other words, religiosity is associated with both the proportion of students who support legalization and the strength with which they express that support. In both sites, non-religious students express higher approval: in Verona, 90.6% of non-religious versus 83.1% of religious students agree or totally agree; in Halle, the difference is even more pronounced, with 88.1% of non-religious and 60.5% of religious students supporting legalization.

A pooled analysis confirms that both national context and religious affiliation are significantly associated with variation in responses (both *p* < 0.001). In addition, religiosity shows a significant association with response patterns, while site has a less pronounced effect.

Compared with the preceding item on ethical justification (Q 4.2), these findings reveal parallel moral and legal trends. Students in Verona articulate clearer and more assertive normative positions: 86.8% (*n* = 151) agree or totally agree with the legalization of active life termination, and nearly half (46.0%, *n* = 80) choose the most affirmative response (*“totally agree”*). In Halle, support is also high (75.0%, *n* = 60) but distributed more evenly across moderate categories, 58.8% agree and only 16.3% totally agree, while 8.8% (*n* = 7) remain undecided.

### Medically assisted suicide: ethical judgment on prescribing drugs (Q 5.2) (Table 10)

**Table 10:** Medically Assisted Suicide (Q 5.2): ethical judgment on prescribing drugs. Ethical judgment on prescribing drugs, by site and religious affiliation. Data are presented as the number of students

		religion		
		no	yes	*p*-value
Halle	**totally disagree**	13	4	0.049
	**not agree**	19	23	
	**undecided**	8	6	
	**agree**	0	4	
	**totally agree**	1	1	
		*p* = 0.053		
Verona	**totally disagree**	31	14	0.001
	**not agree**	42	40	
	**undecided**	11	22	
	**agree**	0	10	
	**totally agree**	1	3	
		*P* < 0.001		

When asked whether a physician providing a patient with access to drugs to end their life is *“never ethically justified”* most students in both Verona and Halle disagree, indicating that they consider MAS ethically acceptable under certain circumstances.

In Verona, 38.3% of students disagree and 25.9% totally disagree, for a combined 64.2% (*n* = 112) who reject the statement. 19.0% are undecided, while 8.0% agree or totally agree, expressing opposition to the practice.

In Halle, 53.2% disagree and 21.5% totally disagree, for a total of 74.7% (*n* = 59) who reject the statement. 17.7% are undecided, and 7.6% agree or totally agree. While the overall pattern is similar across sites, Halle students show a slightly higher level of ethical acceptance of MAS.

No significant difference emerges between the two sites (*p* = 0.873), suggesting comparable ethical orientations across national contexts. Religiosity, however, significantly influences responses overall (*p* = 0.001).

In Halle, religious affiliation affects the *detailed distribution* of answers (item-level *p* = 0.049) but does not significantly change the *overall balance* between agreement and disagreement (*p* = 0.053). In other words, religious and non-religious students in Halle hold broadly similar views but differ in how strongly they express them.

In Verona, by contrast, religion significantly is associated with both the *overall stance* (*p* = 0.001) and the *pattern of item-level responses* (*p* = 0.001). This means that religiosity shapes not only *whether* students find MAS ethically acceptable but also *how firmly* they express this view.

Specifically, in Verona, 85.9% of non-religious students disagree or totally disagree with the statement, thus considering assisted prescription ethically acceptable, compared with 60.7% of religious students.

In Halle, the corresponding proportions are 78.0% among non-religious and 71.1% among religious students, indicating a smaller but similar trend.

A pooled analysis, combining data from both cohorts, confirms that religiosity, but not site, significantly predicts variation in ethical judgment (*p* < 0.001). In other words, across contexts, being religious or non-religious is more strongly associated with variation in responses than national context.

## Discussion

The present study, conducted on two cohorts of fifth-year medical students from the Universities of Verona and Halle, provides a comparative overview of their knowledge and ethical attitudes toward end-of-life practices. Although not all eligible students participated, particularly in Halle, the results show substantial demographic homogeneity between the two groups, with a predominance of female participants and negligible age differences.

The sample is broadly divided between students who identify as religious and those who identify as atheist or agnostic, with an overall similar distribution across the two sites. Among religious students, Catholics clearly predominate in Verona, while in Halle Catholics remain the main group, although a substantial proportion of Protestant students is also present. Overall, this distribution reflects the respective cultural contexts, in which historically rooted religious influences are still evident. However, the considerable proportion of atheist or agnostic students in both cohorts suggests that, among younger populations, religious affiliation no longer constitutes a majority identity marker, but is instead situated within a broader context of increasing pluralism and secularisation.

Personal experience with end-of-life situations is significantly more frequent among students in Halle, both in family contexts and during clinical training. In both cohorts the emotional intensity associated with such experiences remains high.

The results show that both ADs and PC are broadly considered ethically permissible across cohorts. These convergent evaluations should be interpreted in light of the strong normative alignment between ethical principles of autonomy and their legal institutionalization in both Italy and Germany.

With regard to ADs, this consensus reflects, on the one hand, the adherence in both Italy and Germany to the ethical principle of patient autonomy, expressed either directly through informed consent or refusal, or in advance through advance directives, thereby recognising freedom of treatment as the right to decide whether to accept or refuse medical interventions. On the other hand, this convergence is consistent with the fact that ADs have acquired a legally binding status in both countries, thereby further consolidating their clinical legitimacy.

The near-unanimous endorsement of PC observed in the study can be interpreted as an example of convergence between the bioethical paradigm of the sanctity of life and the secular paradigm of quality of life. The former supports PC as an expression of a continuum of care that accompanies the person when the disease is no longer curable, focusing on symptom management and relief of suffering, and thereby avoiding requests for hastened death. The latter frames PC as an essential means of preserving quality of life even in its final stages, in accordance with the principle of self-determination and the possibility of defining goals of care that are proportionate to the patient’s principles, will, and needs [39].

Regarding WLST, within the Italian context there remains a stronger caution, even when treatments are clinically inappropriate. Such an attitude may be consistent with a cultural and educational context in which Christian-Catholic theology and the bioethical paradigm of the sanctity of life have historically played an important role [40, 41], and which is still reflected in contemporary Italian bioethics debates on end-of-life practices [42, 43].

Even more pronounced differences, however, emerge regarding LTTD and MAS, where not only the overall levels of acceptance differ but also the distribution and intensity of moral positions. Students in Verona exhibit a more permissive and polarized orientation, with a clear majority expressing ethical and legal acceptance of such practices. By contrast, students in Halle adopt more moderate and nuanced positions, characterized by a higher proportion of “undecided” responses and fewer extreme judgments.

In both cohorts, religiosity nevertheless remains a key discriminating factor: religious students show a lower propensity to accept LTTD and MAS, both ethically and legally, revealing a stable and coherent association between religious affiliation and moral orientation.

At first glance, it may appear counterintuitive that Italian students, coming from a predominantly Catholic cultural environment, long shaped by the bioethical paradigm of the sanctity of life, exhibit greater ethical acceptance of MAS than their German peers. However, several cultural, educational, and psychological explanations may help clarify this apparent paradox. Overall, these findings can be interpreted within a broader framework of normative enculturation in medical education, whereby ethical judgements are not only the product of individual moral reasoning, but are progressively shaped through the interaction of legal structures, cultural narratives, and clinical training environments. Within this framework, differences across cohorts may also reflect varying degrees of internalization of national legal norms, suggesting that legal permissibility structures do not merely regulate practice but actively shape ethical intuitions in medical trainees.

### Cultural affiliation versus internalized religiosity

In Italy, Catholicism often represents a cultural affiliation rather than a fully internalized or practised faith [44]. Several sociological studies indicate that many young Italians identify as Catholic while displaying low levels of religious participation and limited adherence to the Church’s moral teachings on bioethical matters [45, 46]. In this sense, religion operates primarily as a collective identity framework rather than as an individual normative guide. This may explain why, even in a predominantly Catholic context, the ethical attitudes of medical students appear relatively secularized and more receptive to liberal or autonomy-oriented perspective on the right to die [47, 48].

### The influence of public debate and media context

Over the past two decades, end-of-life issues have become the subject of intense and wide-ranging public and legal debate in both Italy and Germany, with high media coverage in both contexts. In Italy, famous cases such as those of Piergiorgio Welby, Eluana Englaro, and Fabiano Antoniani have profoundly influenced public opinion, framing the right to die as a question of dignity and personal autonomy rather than mere legal permissibility [49, 50]. In 2021, more than 1.2 million signatures were collected in support of a national referendum on euthanasia, although the vote was later blocked on procedural grounds [51, 52]. Moreover, since Constitutional Court ruling no. 242 (2019), MAS has become de facto permissible within the national health system, despite the absence of specific legislation.

In Germany, the 2020 Federal Constitutional Court ruling declaring Sect. 217 StGB unconstitutional similarly generated extensive public discussion and media attention, with debates involving medical associations, religious groups, and civil society organizations [53, 54].

However, the framing and tone of these debates may differ between the two contexts. In Italy, public discourse has been heavily shaped by individual narratives of suffering and calls for compassionate law reform, emphasizing emotional and symbolic dimensions of dignity and self-determination. In Germany, where there is limited knowledge of MAS among the population [55], the debate has been more explicitly structured around constitutional principles, historical memory, and procedural safeguards, reflecting both legal traditions and the enduring influence of historical trauma.

These differences in the character of public debate, rather than its intensity may be associated with the different response patterns observed in the two cohorts, with students in Verona showing a higher proportion of extreme responses and students in Halle a greater use of intermediate response options.

### Differences in medical and bioethical education

Bioethics education in both Italian and German medical schools increasingly includes modules addressing informed consent, advance directives, and the right to self-determination, often emphasizing patient-centred care [56, 57]. While formal curricular content may not differ substantially between the two contexts, other factors may shape how students engage with end-of-life ethics.

One possible interpretative framework for the observed differences may relate to historical memory and its potential role in shaping moral discourse. In Germany, the historical association of the term “euthanasia” with Nazi crimes has been widely discussed in the literature and may still be reflected in contemporary ethical and linguistic sensitivities. The persistent avoidance of the word in both medical and legal discourse could illustrate a collective effort to maintain critical distance from that past, potentially fostering heightened caution and deliberative restraint when discussing practices that involve hastening death. However, this remains a plausible interpretation rather than a demonstrated causal relationship, and further research would be needed to establish whether and how historical memory is explicitly transmitted through medical education or professional socialization. From this perspective, medical curricula should not be understood as neutral spaces of ethical transmission, but as contexts in which legal norms, cultural expectations, and clinical experiences jointly contribute to shaping students’ moral frameworks in end-of-life decision-making. This highlights the need for structured bioethics education that explicitly integrates comparative legal perspectives and reflective engagement with clinical experience. More broadly, these findings suggest that bioethics education functions as a site of normative formation rather than mere knowledge transfer, where moral uncertainty, value pluralism, and cross-jurisdictional legal differences are continuously negotiated and internalized during professional development. From a practical point of view, these findings support the need for strengthening structured bioethics education within undergraduate medical curricula. In particular, greater emphasis should be placed on the integration of comparative legal frameworks into teaching on end-of-life decision-making. In addition, reflective learning based on direct clinical exposure to end-of-life care should be further developed, as experiential engagement appears to play a relevant role in shaping ethical reasoning. Such approaches may contribute to better preparing future physicians to navigate moral uncertainty and value pluralism in clinical practice.

## Conclusion

Overall, the comparative analysis indicates that, despite a shared endorsement of core ethical principles, such as the centrality of the person, decision-making autonomy, and the value of palliative care, significant differences persist in students’ moral evaluations of MAD. These differences reflect both broader national cultural frameworks and the individual influence of religiosity.

Crucially, the most meaningful finding lies not in the average distance between the two cohorts but in the internal configuration of their responses: more polarized and assertive among students in Verona, more gradual and deliberative among students in Halle. This pattern suggests that medical education, together with cultural exposure and personal belief systems, shapes not only *what* future physicians think about end-of-life ethics but *how* they think, whether through value affirmation or through deliberative reasoning.

The convergence in recognizing the ethical and clinical value of ADs and PC, coupled with the divergence concerning MAD, delineates an ethical landscape grounded in a shared respect for human dignity and the duty to accompany patients through dying, yet open to pluralism in moral justification and professional conscience.

### Limitations

The findings presented in this article should be interpreted as the outcome of an exploratory study based on a relatively small and selective sample of medical students. The results should be viewed as indicative rather than definitive. Findings cannot be generalized beyond these specific cohorts because they reflect attitudes among fifth-year students in two mid-sized European universities with Catholic/Protestant cultural influences, not representative of all medical students, practicing physicians, or other countries. This study is affected by convenience sampling bias, as participants may have been overrepresented by motivated/available individuals. Moreover, the responses collected reflect students’ knowledge, attitudes, and perceptions at a specific stage of their academic training and may not necessarily predict future professional behavior or ethical decision-making in clinical practice. Cultural background, curricular differences, and personal experiences with end-of-life care may also have influenced the responses, introducing potential sources of variability beyond the variables formally assessed. Future research should involve larger, more heterogeneous cohorts (ideally including students from diverse universities, healthcare disciplines, and cultural backgrounds) to validate and expand these preliminary findings. Furthermore, multivariate models to control for confounding variables would clarify how training, experience, and moral beliefs interact in shaping end-of-life ethical reasoning.

## Supplementary Information

Supplementary Material 1.

## Data Availability

Data are available on request.
