# Supplementary material for: Knowledge and perception of end-of-life bioethics among medical students. A comparative study between the University of Verona (Italy) and the University of Halle (Germany)
Source: BMC Med Ethics. 2026 Jul 17;27:141. doi: 10.1186/s12910-026-01507-2 (PMC13386620; doi:10.1186/s12910-026-01507-2)
Supplement: Supplementary file 1 — Supplementary Material 1. [file 12910_2026_1507_MOESM1_ESM.doc]

**Knowledge and Perception of End-of-Life Bioethics among Medical Students.**

**A Comparative Study between the University of Verona (Italy) and the University of Halle (Germany)**

**Questionnaire**

*(In the German-language questionnaire, the word “Euthanasia” will be translated with "Ending of life on request")*

**Accompaniment to death**

- 1. Have you ever accompanied a dying person?

□ Yes

□ No

1.2 If you have answered question 1 with “Yes”: how intensive would you describe this experience?

Experience in the family or with friends

□ little intensive

□ intensive

□ very intensive

Experience in internship of medical care

□ little intensive

□ intensive

□ very intensive

Other situations (what kind of situations?): ___________________________________

□ little intensive

□ intensive

□ very intensive

1.3 As a medical student, have you ever been asked about assisted death (euthanasia or medically assisted suicide) by a patient?

□ Yes

□ No

**Advance Directives**

2.1 Are they legally binding in your Country?

□ Yes

□ No

□ I don’t know

2.2 Do you believe they are a valid tool for expressing one’s will for when one is no longer capable of doing so?

□ Yes

□ No

2.3 Do you think they are a useful tool for physicians when it comes to making decisions regarding end-of-life care?

□ Yes

□ No

**Withdrawal of Life-Sustaining Treatments and Palliative Care**

3.1 Do you believe that in some clinical situations, medical intervention should be withdrawn even if it is a life-saving treatment?

□ Yes

□ No

3.2 According to your knowledge, the will of a patient with decisional capacity to Withhold or to Withdraw a Life-Sustaining Treatment, must be always legally granted?

□ In all cases.

□ In certain cases.

□ Never.

3.3 According to your perception: is palliative care integral part of medical practice or not?

□ Palliative Care practices are an integral part of medical practice.

□ Palliative Care practices are still not an integral part of medical practice.

**Euthanasia**

4.1 According to your knowledge, is it legal in your country?

□ Yes

□ No

□ I don’t know

4.2 Euthanasia is never ethically justified.

□ I totally agree

□ I disagree

□ I am undecided

□ I agree

□ I totally agree

4.3 There are situations where euthanasia should be legal.

□ I totally agree

□ I disagree

□ I am undecided

□ I agree

□ I totally agree

**Medically Assisted Suicide**

5.1 According to your knowledge, is it legal in your country?

□ Yes

□ Only in certain cases

□ No

□ I don’t know

5.2 Prescribing drugs for the purpose of patient’s suicide is never ethically justified.

□ I totally agree

□ I disagree

□ I am undecided

□ I agree

□ I totally agree

5.3 There are situations where prescribing drugs for the purpose of patient’s suicide should be legal.

□ I totally agree

□ I disagree

□ I am undecided

□ I agree

□ I totally agree

**Educational training**

6. With respect to your undergraduate medical training - how well prepared to consider ethical questions concerning the ending of life?

□ Very good

□ Good

□ Satisfactory

□ Sufficient

□ Deficient

□ Insufficient

**Personal Data**

Gender

□ female

□ male

Religion

□ Protestant

□ Roman-Catholic

□ Muslims

□ No congregation/church

□ Other Religion

Age

________years
